# Genetic algorithm for the optimization of features and neural networks in ECG signals classification

**DOI:** 10.1038/srep41011

**Published:** 2017-01-31

**Authors:** Hongqiang Li, Danyang Yuan, Xiangdong Ma, Dianyin Cui, Lu Cao

**Affiliations:** 1 Tianjin Key Laboratory of Optoelectronic Detection Technology and Systems, School of Electronics and Information Engineering, Tianjin Polytechnic University, Tianjin 300387, China; 2 Tianjin Chest Hospital, Tianjin 300222, China

## Abstract

Feature extraction and classification of electrocardiogram (ECG) signals are necessary for the automatic diagnosis of cardiac diseases. In this study, a novel method based on genetic algorithm-back propagation neural network (GA-BPNN) for classifying ECG signals with feature extraction using wavelet packet decomposition (WPD) is proposed. WPD combined with the statistical method is utilized to extract the effective features of ECG signals. The statistical features of the wavelet packet coefficients are calculated as the feature sets. GA is employed to decrease the dimensions of the feature sets and to optimize the weights and biases of the back propagation neural network (BPNN). Thereafter, the optimized BPNN classifier is applied to classify six types of ECG signals. In addition, an experimental platform is constructed for ECG signal acquisition to supply the ECG data for verifying the effectiveness of the proposed method. The GA-BPNN method with the MIT-BIH arrhythmia database achieved a dimension reduction of nearly 50% and produced good classification results with an accuracy of 97.78%. The experimental results based on the established acquisition platform indicated that the GA-BPNN method achieved a high classification accuracy of 99.33% and could be efficiently applied in the automatic identification of cardiac arrhythmias.

An electrocardiogram (ECG) is a complete representation of the electrical activity of the heart on the surface of the human body, and it is extensively applied in the clinical diagnosis of heart diseases^1^,^2^,^3^. Many studies have developed arrhythmia recognition approaches that utilize automatic analysis and diagnosis systems based on ECG signals^4^,^5^,^6^,^7^, in which feature extraction and classification are particularly important for the analysis and diagnosis of cardiac diseases. Numerous techniques for classifying ECG signals have been proposed in recent years. A modified artificial bee colony algorithm was established for ECG heartbeat classification to classify time domain features, and good results were achieved^8^. An automatic ECG classification method using BPNN combined with wave characteristics was presented to distinguish and diagnose heart diseases^9^. A technique based on time domain features and support vector machine was applied to an ECG dataset to analyze and classify cardiac arrhythmias^10^. Although ECG features in the time domain can be easily obtained, these features rely excessively on waveform detection and are easily affected by noise. Transform methods are also widely applied in feature extraction because of their good time–frequency property. Discrete biorthogonal wavelet decomposition was utilized for extracting ECG features, and a radial basis function neural network was used for ECG classification^11^. A combined neural network model was designed for the classification of ECG beats; this model was trained and tested using discrete wavelet transform on the extracted features^12^. Wavelet algorithm was applied in extracting features, and fuzzy neuro learning vector quantisation (FLVQ) was used as the classifier for arrhythmia beats^13^. An ECG beat classification method was presented, wherein discrete cosine transform converted RR intervals and random forest was used as the classifier^14^. Feature extraction using discrete wavelet transform and multiclass support vector machines was employed for the classification of four types of ECG beats^15^. Moreover, combining several methods is a common strategy in ECG feature extraction and classification. Features obtained by independent component analysis, together with the use of the RR interval as the feature vector, were entered into neural networks for ECG beats classification^16^. Cross-correlation was utilized as a formidable feature extraction tool and the least squares support vector machine (LS-SVM) was employed as an automated ECG beat classifier^17^. A combined method based on stacked generalisation was proposed for classifying ECG beats; in this method, multilayer perceptron classifiers were utilized as the base classifiers trained by the back propagation algorithm^18^. Higher-order statistics (HOSs) of ECG signals and three time interval features were fed as features into a bee algorithm–radial basis function classifier to classify five types of ECG beats^19^. HOSs of WPD coefficients were used as the features for ECG heartbeats classification, and the obtained features were classified by a k-nearest neighbor classifier^20^.

In the present study, we used the WPD combined with the statistical method (WPD-statistical method) to extract useful features. Then, we applied the GA-BPNN method to filter the extracted features and classify the six types of ECG signals. Prior to feature extraction, a method based on the improved threshold of the lifting wavelet was applied to remove the noise from ECG signals in preprocessing^21^,^22^. Then, GA-BPNN method was employed to select representative features and optimize the BPNN classifier. The filtered features were inputted into the optimized BPNN classifier for classification. In this study, the ECG signals derived from the MIT-BIH arrhythmia database^23^ were classified into six categories, namely, normal beat (N), left bundle branch block beat (L), right bundle branch block beat (R), atrial premature beat (A), paced beat (P), and premature ventricular contraction (V). We also constructed an experimental platform of ECG acquisition to supply six types of ECG signals for verifying the effectiveness of the proposed method. Figure 1 presents the overall block diagram of the proposed method for ECG signal classification.

## Results

### Data sets from MIT-BIH database

Complete experimental analysis was conducted to evaluate the performance of the proposed approach. In this study, six types of ECG signals were obtained from the MIT-BIH arrhythmia database, and the sampling rate was 360 Hz^23^. We used a segment of 1000 points from each type containing relevant ECG signal information and selected 360 samples for ECG classification. The sampling data collected in this study from the MIT-BIH arrhythmia database are listed in Table 1.

### Feature extraction based on WPD-statistical method

**Figure 2:**
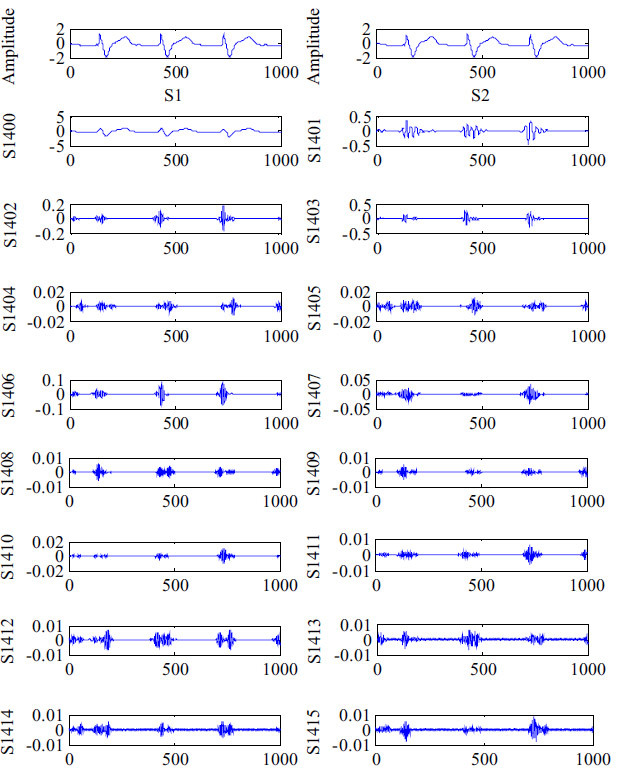
Results of ECG signal decomposition using WPD. S1 and S2 refer to the original and preprocessed ECG signals, respectively. S1400–S1415 represent the 16 WPCs.

In this study, we extracted feature vectors by using WPD-statistical method. We selected the db6 wavelet as the mother wavelet. The ECG signal segments were decomposed into 4 levels as shown in Fig. 2. Then, by employing the statistical method, 16 wavelet packet coefficients (WPCs) in the fourth level of WPD were calculated to obtain the ECG features. Each ECG signal segment contained 16 WPCs. As such, the feature matrix consisted of 48 (16 × 3) dimensions, and the as-extracted features were used for ECG feature selection and classification.

### Feature selection and the BPNN structure optimization using GA

**Figure 3:**
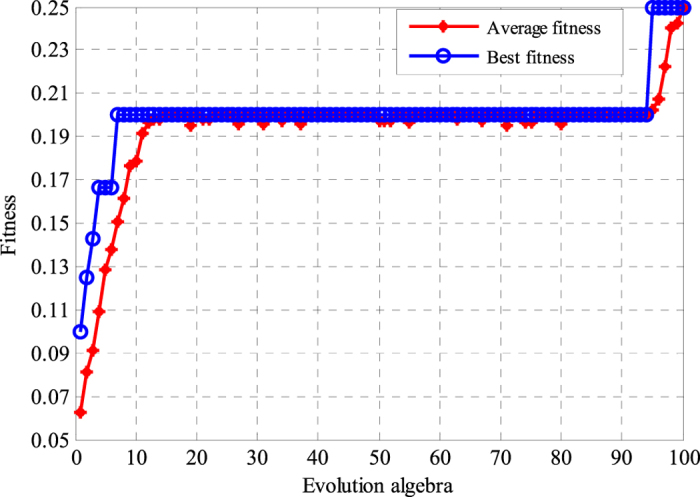
Fitness curve of GA. Average fitness and best fitness were gradually increased via a series of iterations. When the evolution algebra was 100, the average fitness and the best fitness reached the maximum value; that was, the sum of square error of the test set obtained the least value.

After extracting the features using the WPD-statistical method, we obtained a 180 × 48 training feature matrix and a 180 × 48 testing feature matrix. To improve the classification efficiency and decrease calculation, redundant features were essential to remove. Therefore, The GA-BPNN method was employed to filter representative features for ECG signals classification. Furthermore, the initial weights and biases of the BPNN were optimized by GA because their randomness would affect the testing result. The parameters of GA were set as follows: the number of individual was 48; the population size was 20; and the maximum generation was 100. The fitness curve of GA is illustrated in Fig. 3. After a series of iterations, the average fitness and the best fitness were gradually improved, and a set of input arguments were filtered by GA optimization. After 100 iterations, the filtered feature numbers were as follows: 1, 5, 8, 10, 12, 13, 14, 17, 18, 20, 22, 23, 26, 27, 29, 30, 32, 33, 34, 35, 36, 39, 40, 45, and 46. The dimensions of the feature sets were reduced to approximately 50% by utilizing GA.

### The ECG classification results of the BPNN classifiers

The filtered feature sets were inputted into the optimized BPNN classifier. A 180 × 25 feature matrix was used as the training set to train the optimal BPNN model, and a 180 × 25 feature matrix was utilized as the testing set for classification and prediction. The training parameters of the BPNN classifier used in this study were as follows: The momentum back propagation algorithm was applied to train the BPNN classifier. The structure of the BPNN classifier consisted of one input layer, two hidden layers and one output layer. Logistic functions were used in the hidden layers. A total of 48 input layer nodes, 50 hidden layer nodes and 6 output layer nodes were set. The maximum iteration was 1000 epochs, the minimum error goal was set as 0.01 and the learning rate was 0.05.

**Figure 4:**
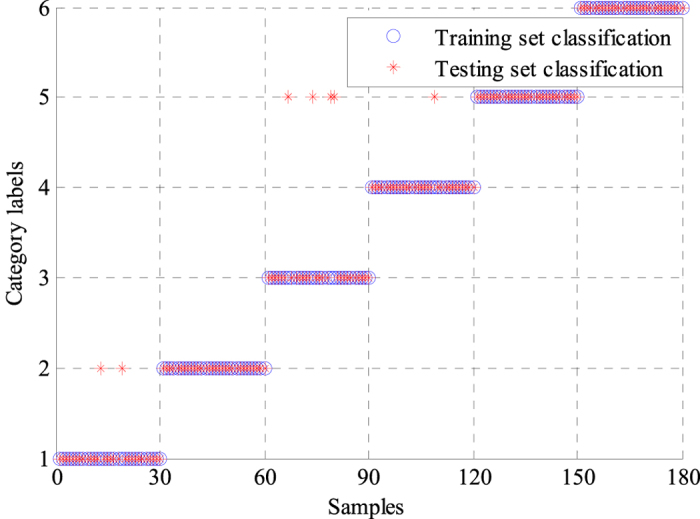
Classification results of the single BPNN classifier. The classification accuracy of the training set was 100%. Six types of ECG signals in the testing set had different classification results. Samples of L, V and A were correctly classified. Two samples of N were classified to L. Four samples of R and one sample of P were classified into V. Accordingly, the classification accuracy of N, L, R, P, V, and A were 93.33%, 100%, 86.67%, 96.67%, 100%, and 100%, respectively.

**Figure 5:**
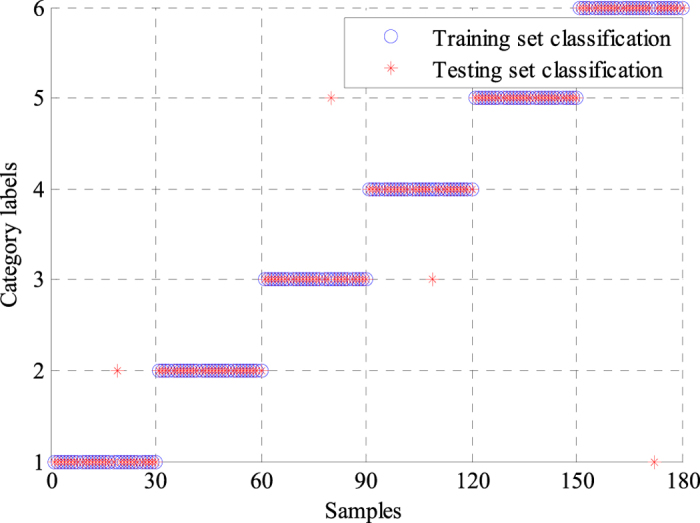
Classification results of the GA-BPNN classifier. The classification accuracy of the training set was 100%. In testing set classification, there were different results in six types of ECG signals. Samples of L and V were correctly classified. One sample of N was wrongly classified into L. One sample of R was categorized into V. One sample of P was classified into R. One sample of A was wrongly categorized into N. Thus, the classification accuracy of N, L, R, P, V, and A were 96.67%, 100%, 96.67%, 96.67%, 100%, and 96.67%, respectively.

**Figure 6:**
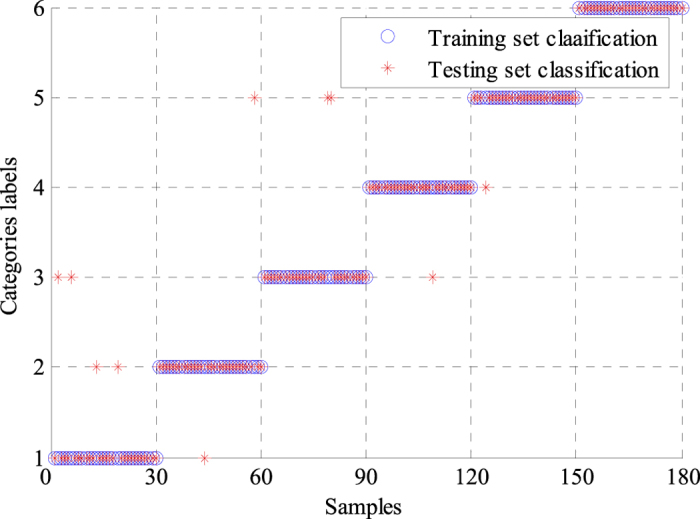
Classification results of the SVM classifier.

**Figure 7:**
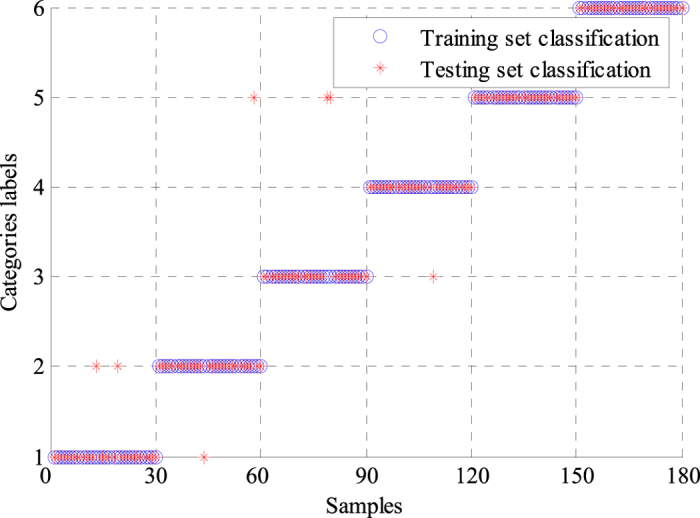
Classification results of the GA-SVM classifier.

We also used a single BPNN classifier to classify the features extracted by the WPD-statistical method, and the results were compared with those obtained by the GA-BPNN method. The average modeling time of the optimized BPNN classifier was only 3.1652 s, whereas that of the single BPNN model was 8.0231 s, indicating that the modeling time was significantly reduced by GA optimization. The classification results of the two classifiers are presented in Figs 4 and 5. Labels 1 to 6 represent N, L, R, P, V and A. Labels ‘°’ and ‘*’ denote the training and testing sets, respectively. As shown in Figs 4 and 5, the training sets of the two classifiers were classified correctly. Only four samples of the testing set were incorrectly classified by the GA-BPNN method, whereas the single BPNN classifier incorrectly classified seven samples of the testing set. Four statistical indices, namely, sensitivity (*Se*), specificity (*Sp*), positive predictive value (*PPV*) and classification accuracy (*A*_*CC*_) were calculated for analysis and comparison to evaluate the performance of the two classifiers better. These statistical indices were defined in following equations:


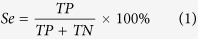



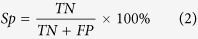



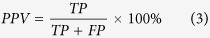



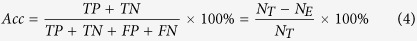


where *TP, TN, FP* and *FN* denote true positive, true negative, false positive and false negative, respectively. *N*_*T*_ represents the number of correctly classified ECG signals, whereas *N*_*E*_ indicates the number of incorrectly classified ECG signals. The performance statistics of the two classifiers are shown in Tables 2 and 3. The GA-BPNN method achieved a higher classification accuracy of 97.78% than the classification accuracy of 96.11% obtained by the single BPNN classifier, which suggested the proposed method based on the GA-BPNN classifier could lead to better classification results. The average sensitivity was greatly improved from 96.11% to 97.86% when the classifier and features were optimized by using GA. The average specificities of the two classifiers were very similar. The average positive predictive value was also raised from 96.58% to 97.81%. For making comparisons with different classifiers, we also used SVM and genetic algorithm-support vector machine (GA-SVM) to classify the same ECG features extracted by WPD-statistical method. Figures 6 and 7 show the classification results of SVM and GA-SVM, individually. The comparison results of different classifiers are shown in Table 4. We can see clearly from Table 4 that the GA-BPNN classifier achieved superior performance over other classifiers while using the same data of ECG signals.

### Experimental results from the ECG acquisition platform

**Figure 8:**
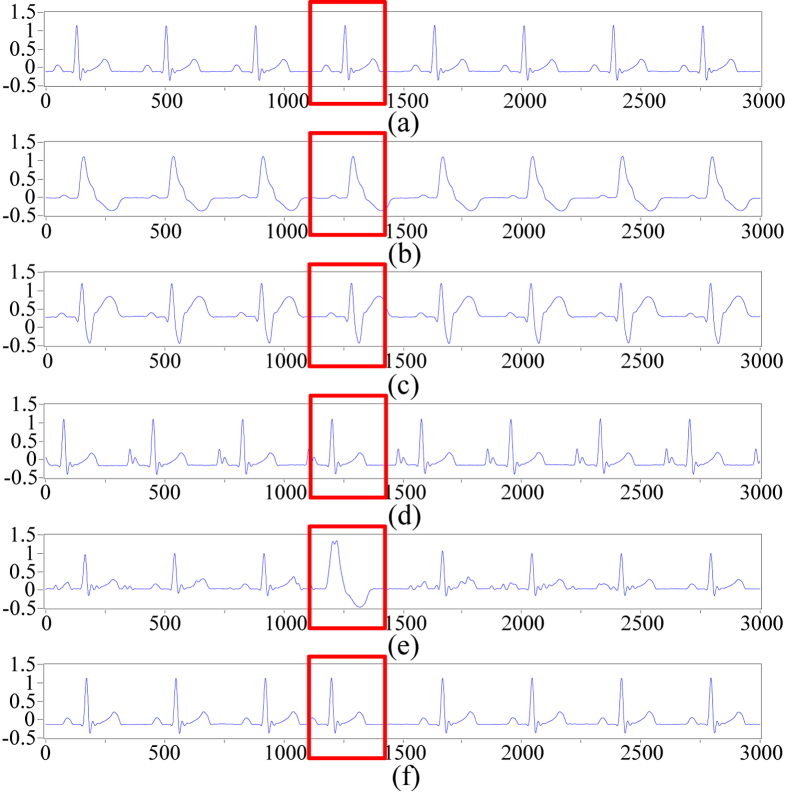
Six categories of ECG signals collected with the experimental platform. Each category of ECG signal is highlighted in the red dashed boxes. (**a**) N, (**b**) L, (**c**) R, (**d**) P, (**e**) V and (**f** ) A.

**Figure 9:**
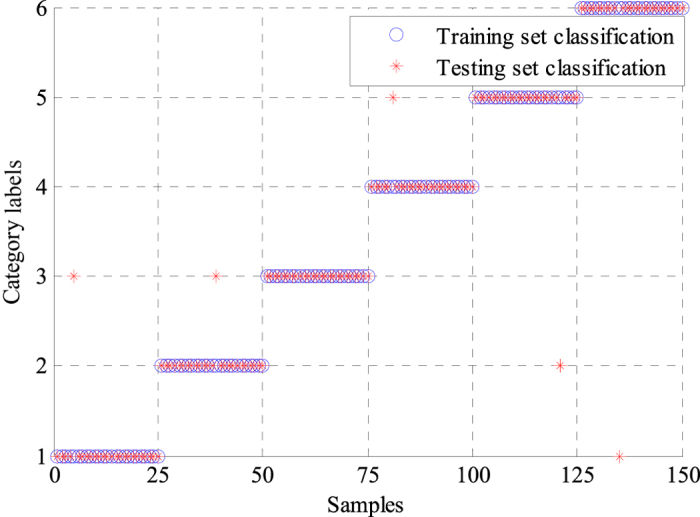
Classification results of the single BPNN classifier using the collected ECG signals.

**Figure 10:**
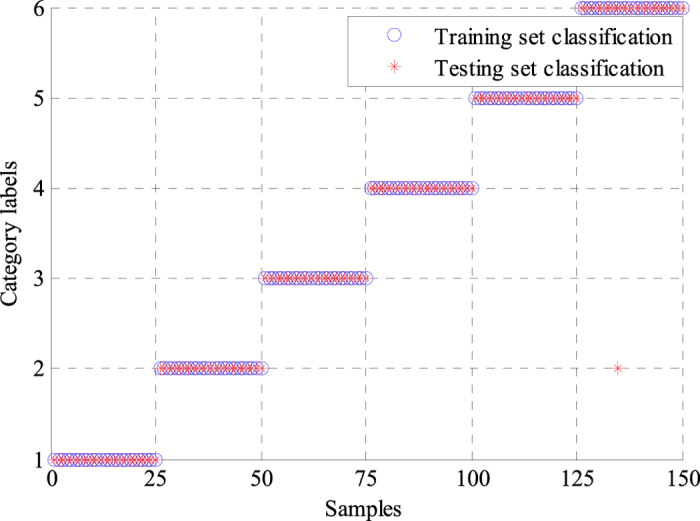
Classification results of the GA-BPNN classifier using the collected ECG signals.

A total of 300 samples containing 6 types of ECG signals were collected using the ECG acquisition experimental platform, as shown in Fig. 8, and were inputted into the proposed method for identification. Noise was eliminated from the acquired ECG signals using the method based on the improved half-soft threshold of the lifting wavelet. Then, the WPD-statistical method was applied to extract ECG features after preprocessing. The statistical features of WPCs with 48 dimensions were obtained as ECG features. Thereafter, GA was applied to optimize the features and the neural network for ECG signals identification. The filtered features were classified with the optimal classifier based on GA-BPNN into six categories. We also adopted a single BPNN classifier to recognize the collected ECG signals, and the results were compared with those obtained by the GA-BPNN classifier. Figures 9 and 10 show the classification results of the two classifiers using the ECG data acquired from the FLUKE ProSim 2 vital sign simulator. The single BPNN classifier using the collected ECG signals produced satisfactory classification results, and five samples were incorrectly classified. The GA-BPNN classifier achieved better identification results, and only one sample was incorrectly classified. The statistical performance indicators of the two classifiers were calculated, as shown in Tables 5 and 6. The single BPNN classifier exhibited good performance, with an accuracy of 96.67%, a sensitivity of 96.67%, a specificity of 99.33% and a positive predictive value of 96.77%. However, the GA-BPNN classifier achieved a higher classification accuracy of 99.33% and performed better than the single BPNN classifier in the other three statistical indicators.

### Discussion and Conclusion

As shown in Tables 3 and 6, the proposed method using the ECG data from the MIT-BIH arrhythmia database produced good identification results, with an accuracy of 97.78%, an average sensitivity of 97.86%, an average specificity of 99.54% and an average positive predictive value of 97.81%. The ECG acquisition experimental platform also achieved good experimental classification results, with an average sensitivity of 99.33%, an average specificity of 99.87%, an average positive predictive value of 99.36% and an accuracy of 99.33%. Although the proposed method using the ECG data from the experimental platform achieved excellent identification results, the methods using more samples from the MIT-BIH arrhythmia database also obtained good performance statistics of classification. The main reason is that the FLUKE ProSim 2 vital sign simulator is restricted as the signal source to supply a large amount of ECG signals. Another important reason is that ECG signals might require other features to reflect their characteristic information. Thus, we intend to investigate the algorithm further with regard to feature extraction and classification in the diagnosis of heart disease.

The proposed method was compared with several other methods based on the MIT-BIH arrhythmia database, as shown in Table 7. Nine waveform features (RR interval, P height, R height, heart rate, QT interval, ST interval, QRS width, corrected QT interval and PR interval) were fed into the LIBSVM classifier for classifying the ECG signals into five categories, and an accuracy of 95.21% was obtained^10^. Jatmiko *et al*. extracted features using wavelet transform and used FLVQ to classify ECG signals into five classes with an accuracy of 95.50%^13^. HOSs of ECG signals combined with three time interval features were extracted, and the hybrid bee algorithm–radial basis function (RBF-BA) technique was used to classify the five types of ECG signals with an accuracy of 95.79%^19^. A cross-correlation approach was used, in which the cross-spectral density information in the frequency domain was utilised to extract features, and the LS-SVM classifier classified the features of ECG beats into three categories with an accuracy in the range of 95.51% to 96.12%^17^. The proposed method classified six types of ECG signals with a high accuracy of 97.78%. As shown in Table 7, our proposed method can achieve more categories with a satisfactory identification result. The proposed method requires only small groups of representative datasets as feature sets and uses the optimized BPNN as the classifier using GA, thereby considerably improving the classification performance.

In this study, a novel method based on the GA-BPNN was designed for optimizing features and neural networks to classify six types of ECG signals. A total of 360 groups of datasets derived from the MIT-BIH arrhythmia database were preprocessed to eliminate noise interference. Then, the WPD-statistical method was employed for feature extraction. We obtained 48 features of each ECG sample by calculating the singular value, the standard deviation and the maximum value of the 16 WPCs in each ECG segment. Thereafter, 25 representative features were filtered and fed into the optimal BPNN classifier using the GA-BPNN method. The presented method with MIT-BIH arrhythmia database achieved a feature reduction of nearly 50% and yielded the classification accuracy of 97.78%, a sensitivity of 97.86%, a specificity of 99.54% and a positive predictive value of 97.81%. Moreover, an ECG signal acquisition experimental platform was constructed to collect ECG signals to demonstrate the utility of the proposed system. The experimental results obtained with the established acquisition platform illustrated that the GA-BPNN method achieved 99.33% accuracy, 99.87% sensitivity, 99.36% specificity and 99.33% positive predictability. Therefore, the proposed method can achieve high classification accuracy and has promising potential application in the automatic diagnosis of heart diseases.

## Methods

In this section, the proposed framework based on GA-BPNN, experiment setup and three main mathematical foundations consist of WPD, GA and BPNN, are listed to support the presented study.

### The proposed framework based on genetic algorithm-back propagation neural network

Due to WPD has an accurate frequency resolution, we decompose the ECG signals using WPD up to level four in this study. The WPCs in fourth level of each signal segment are calculated to obtain ECG features. The statistical information of WPCs are used as features rather than the HOSs of WPCs to represent ECG signals because these features are simple to calculate and also contain a lot of detailed frequency characteristics, which differs from the worked method by Kutlu and Kuntalp^20^. The following statistical features of the WPCs are calculated to represent the information of ECG signals:

1. The singular value of the WPCs in each segment.
2. The standard deviation of the WPCs in each segment.
3. The maximum value of the WPCs in each segment.

After feature extraction, the main technique of the proposed method based on GA optimization is employed to reduce the dimensions of the feature sets and optimize the BPNN’s weights and biases. The concrete designing steps of the proposed framework are devised as follows:

1. A single BPNN is established for comparison with the GA-BPNN model.
2. An initial population of N individuals is created. Binary coding is used, and N is considered equal to the dimensions of the feature sets.
3. The fitness value is calculated based on the sum of square error (SSE) of the test set, as shown in Equation (5), where *f*(*X*) is the fitness value, 
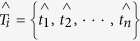
 is the value of predicted test set, and *T*_*i*_ = {*t*_*1*_, *t*_*2*_, …, *t*_*n*_} is the actual test set. *n* denotes the test set samples. Individual with high fitness have a high probability of being reserved.
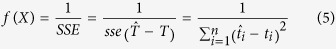
4. Three genetic operators are employed to generate new solutions. Roulette wheel selection is applied to select parent solutions. Offspring are produced using single point crossover, and these offspring are mutated by single point mutation for offering variation.
5. The fitness value of the newly generated individuals is updated.
6. Steps 3 to 5 are repeated until the stop condition (maximum generation is reached) is satisfied. Consequently, the optimal solutions are the outputs; that is, the most representative features are filtered.
7. The optimal BPNN model is established. On the basis of the results calculated by GA optimization, the filtered features containing the training and testing sets are extracted to re-establish the BPNN model for simulation test and result analysis.

The weights and biases of the BPNN are optimized using GA and each individual fitness value is computed to avoid the randomness of the initial weights and thresholds, both of which affect the fitness calculation. We apply the steps of GA to attain the BPNN model with the optimized weights and biases. The weights and biases of the BPNN are coded by real-number coding. The utilized fitness value is the reciprocal of the mean square error between the expected output and the actual output. Roulette wheel selection, arithmetic crossover, and non-uniform mutation are employed to optimize the weights and biases. The optimal BPNN model is established after a series of iterations, which is terminated when the maximum number of iterations is reached. When the filtered feature sets are inputted into the optimal BPNN classifier, the outputs are automatically classified into the six types of ECG signals.

### Wavelet packet decomposition analyzed in feature extraction

WPD is a useful tool for analyzing and extracting information from ECG signals^20^,^24^. WPD is an extension of wavelet decomposition (WD). The multi-resolution of WD can only decompose the low-frequency parts of a signal. WPD not only has a fine frequency resolution at low frequencies but also has a fine frequency resolution at high frequencies. In the structure of WD, the signals are split into low-frequency contents (approximations) and high-frequency contents (details). The approximations obtained from the first level are divided into new approximation and detail contents. The procedure is then repeated. Hence, WD has poor frequency resolution in high-frequency contents. Different from WD, WPD provides highly detailed signal decomposition and improves signal time–frequency resolution. WPD can attain high separation in the high frequency contents of a signal, which will be useful in the classification. Thus, in this study, we employed WPD to decompose ECG signals and analyze their characteristics.

Many types of mother wavelets exist, such as Haar, Meyer, Daubechies (db), Biorthogonal, Coiflet, and Symlet. The WPCs and classification accuracies of the extracted features from each mother wavelet are compared, and the best feature set is attained by using the db6 wavelet function^20^. Therefore, the db6 wavelet is used as the mother wavelet function to assess WPCs in the proposed method. Four levels of decomposition are applied to obtain more detailed features.

### Fundamental analysis of genetic algorithm

GA, which was invented by Holland^25^, is a type of optimization searching algorithm based on natural evolution and theory of genetics. GA has been widely used in feature selection and neural network structure optimisation^26^,^27^. Combining GA with neural networks can overcome several problems, such as the local limit value and the slow rate of convergence, thereby efficiently increasing the training speed of networks. GA is able to filter and optimize the input features before building the model contains numerous inputs. The first step in general GA is to randomly generate an initial population of individuals. Thereafter, the performance of the population is evaluated by using an individual fitness function. GA operates the genetic constitution of a population through three basic operators, namely, selection, crossover, and mutation. The selection process directly selects the most highly rated individuals from the current population on the basis of the fitness values of every individual. The crossover plays a role in creating new individuals from old ones. Crossover randomly exchanges the information between two parents for generating new individuals. Mutation is a process that randomly selects an individual in the population and alters some values in the individual based on mutation rate. Mutation gives opportunities for creating new individual. When the termination condition is satisfied, the optimal solution will be filtered after a series of iterative calculations with these operators.

### Back propagation neural network analyzed in ECG classification

Artificial neural network (ANN) is widely applied in the application fields of pattern recognition and classification. BPNN is one of the most popular and important networks of ANN^27^,^28^. BPNN has a strong associative memory and prediction capability after training. Thus we choose the BPNN as the classifier for ECG signals classification. The BPNN is a multilayer feed-forward neural network composed of three parts, namely, the input layer, the hidden layer and output layer. *X*_1_, *X*_2_, …, *X*_*n*_ represented the BPNN inputs and *Y*_*1*_, *Y*_*2*_, …, *Y*_*m*_ denoted the BPNN outputs. The network input layer node *n* was based on the dimensions of the feature sets. The output layer node *m* is equal to the number of ECG signal types. No unified standard is available for selecting hidden layer nodes, which are typically determined by assessing the overall accuracy. The BPNN utilized three main activation functions, namely, logistic function, hyperbolic tangent function and identity function. In this study, the logistic function is used in the input and hidden layers, as shown in the following equation:


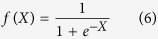


Many learning algorithms are utilized in BPNN, such as gradient descent, momentum back propagation, and Levengerg–Marquardt. The BPNN is trained using the momentum back propagation algorithm because of the high convergence rate and short learning time of this algorithm.

### Experiment setup

**Figure 11:**
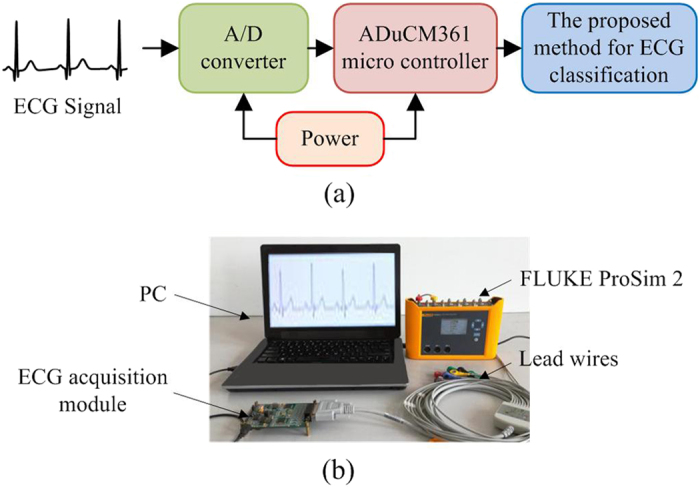
Experimental platform for ECG signal acquisition. (**a**) Block diagram of the experimental platform and (**b**) construction of the experimental platform.

An experimental platform is constructed for ECG acquisition to collect ECG signals for arrhythmia classification to verify the effectiveness of the proposed method for ECG identification. We utilize the FLUKE ProSim 2 vital sign simulator (produced by FLUKE Corp., Everett, WA, USA) as the signal resource to provide six types of ECG signals, and we select the standard I lead ECG in this platform. During ECG signal acquisition, an ADuCM361 microcontroller (Analog Devices, Inc., Norwood, MA, USA) is used to control the A/D converter (Analog Devices, Inc., Norwood, MA, USA) for converting analogue ECG signals into digital signals. The acquired ECG signals are transmitted via a USB cable to a PC and are processed by the proposed method for classification. Moreover, the ECG acquisition module is supplied with power via the USB cable. Figure 11 depicts the diagrams of the proposed ECG acquisition experimental platform.

## Additional Information

**How to cite this article:** Li, H. *et al*. Genetic algorithm for the optimization of features and neural networks in ECG signals classification. *Sci. Rep.* **7**, 41011; doi: 10.1038/srep41011 (2017).

**Publisher's note:** Springer Nature remains neutral with regard to jurisdictional claims in published maps and institutional affiliations.

## Figures and Tables

**Figure 1:**
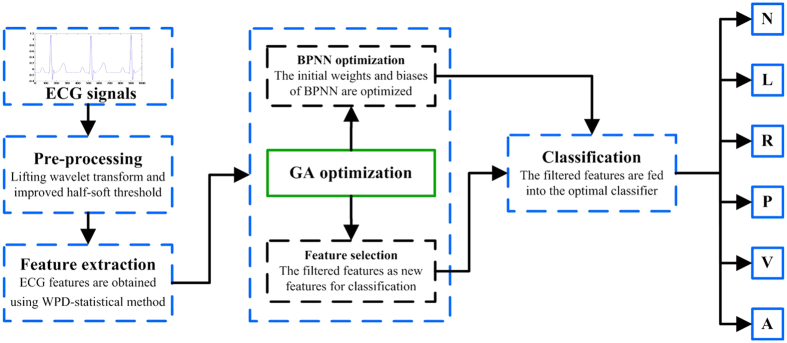
The block diagram of the proposed method for ECG signals classification. The classification method consists of preprocessing, feature extraction, GA optimization and classification. Preprocessing is performed to remove noise from the original ECG signals. Feature extraction is conducted to obtain ECG features using the WPD-statistical method. GA optimization is employed to reduce the feature dimensions and to optimize the weights and biases of BPNN. Classification refers to classifying ECG signals into six types, namely, N, L, R, P, V and A.

**Table 1:** The ECG data is sampled from MIT-BIH database.

Type	MIT-BIH	The training set	The testing set
N	100, 105, 215	30	30
L	109, 111, 214	30	30
R	118, 124, 212	30	30
P	102, 107, 217	30	30
V	106, 223	30	30
A	207, 209, 232	30	30
	Total	180	180

Each type of ECG signals had 30 samples for the training set and 30 samples for the testing set. Samples of N were obtained from records 100, 105 and 215. Samples of L were derived from records 109, 111 and 214. Samples of R were obtained from records 118, 124 and 212. Samples of P were obtained from records 102, 107 and 217. We obtained samples of V from records 106 and 223 and those of A from records 207, 209 and 232.

**Table 2:** The performance statistics of the single BPNN classifier.

Type	*Se*	*Sp*	*PPV*
N	93.33%	100%	100%
L	100%	98.62%	93.75%
R	86.67%	100%	100%
P	96.67%	100%	100%
V	100%	96.62%	85.71%
A	100%	100%	100%
Average	96.11%	99.21%	96.58%
*A*_*CC*_	96.11%		

Six types of ECG signals had different performance in the classification results. A has best performance statistics of sensitivity, specificity and positive predictive value. L performed well with a sensitivity of 100%, a specificity of 98.62% and a positive predictive value of 93.75%. N, R and P had good performance in specificity and positive predictive value, but the sensitivity of N was lower than other types of sensitivities. Moreover, V had poor performance in positive predictive value.

**Table 3:** The performance statistics of the GA-BPNN classifier.

Type	*Se*	*Sp*	*PPV*
N	93.55%	100%	100%
L	100%	99.31%	96.77%
R	96.67%	99.32%	99.67%
P	96.67%	100%	100%
V	100%	99.31%	96.77%
A	100%	99.32%	99.67%
Average	97.86%	99.54%	97.81%
*A*_*CC*_	97.78%		

Six types of ECG signals performed well in the classification results. N and P had good performance in specificity and positive predictive value whereas the sensitivity of N was lower than the sensitivity of P. L and V had the same performance statistics in the classification results. R and A had the same performance in specificity and positive predictive value, but A performed better than R in sensitivity.

**Table 4:** The comparison results of different classifiers.

Classifier	*Se*	*Sp*	*PPV*	*Acc*
SVM	94.45%	98.89%	94.54%	94.44%
GA-SVM	96.11%	99.22%	96.22%	96.11%
BPNN	96.11%	99.21%	96.58%	96.11%
GA-BPNN	97.86%	99.54%	97.81%	97.78%

**Table 5:** The performance statistics of single BPNN classifier with the experimental platform.

Type	*Se*	*Sp*	*PPV*
N	96%	99.2%	96%
L	96%	99.2%	96%
R	100%	98.4%	92.59%
P	96%	100%	100%
V	96%	99.2%	96%
A	96%	100%	100%
Average	96.67%	99.33%	96.77%
*A*_*CC*_	96.67%		

**Table 6:** The performance statistics of GA-BPNN classifier with the experimental platform.

Type	*Se*	*Sp*	*PPV*
N	100%	100%	100%
L	100%	99.2%	96.15%
R	100%	100%	100%
P	100%	100%	100%
V	100%	100%	100%
A	96%	100%	100%
Average	99.33%	99.87%	99.36%
*A*_*CC*_	99.33%		

**Table 7:** Comparison with other methods based on the MIT-BIH arrhythmia database.

Methods	Features	Classifier	Classes	Accuracy
Bhardwaj *et al*.	Waveform features	LIBSVM	5	95.21%
Jatmiko *et al*.	Wavelet transform coefficients	FLVQ	4	95.50%
Ebahimzadeh *et al*.	HOSs and timing interval features	RBF-BA	5	95.79%
Dutta *et al*.	Features based on cross-correlation	LS-SVM	3	95.51–96.12%
Proposed method	Statistical features of WPCs	GA-BPNN	6	97.78%

## References

[b1] Lobodzinski S. S. ECG patch monitors for assessment of cardiac rhythm abnormalities. Prog. Cardiovasc. Dis. 56, 224–229 (2013). 24215754 10.1016/j.pcad.2013.08.006

[b2] Bergfeldt L. Differential diagnosis of cardiogenic syncope and seizure disorders. Heart. 89, 353–358 (2003). 12591858 10.1136/heart.89.3.353 PMC1767616

[b3] Johannesen L. . Wavelet-based algorithm for delineation and classification of wave patterns in continuous Holter ECG recordings. Comput. Cardiol. 37, 979–982 (2010). PMC3139228 21779544

[b4] Zhao Z., Yang L., Chen D. & Luo Y. A human ECG identification system based on ensemble empirical mode decomposition. Sensors-Basel. 13, 6832–6864 (2013). 23698274 10.3390/s130506832 PMC3690084

[b5] Valenza G., Citi L., Lanatá A., Scilingo E. P. & Barbieri R. Revealing real-time emotional responses: a personalized assessment based on heartbeat dynamics. Sci. Rep. 4, 1–13 (2014). 10.1038/srep04998 PMC4028901 24845973

[b6] Valenza G. . Inhomogeneous point-processes to instantaneously assess affective haptic perception through heartbeat dynamics information. Sci. Rep. 6, 1–14 (2016). 27357966 10.1038/srep28567 PMC4928096

[b7] Luz E. J. D. S., Schwartz W. R. & Menotti D. ECG-based heartbeat classification for arrhythmia detection: a survey. Comput. Meth. Prog. Bio. 127, 144–164 (2016). 10.1016/j.cmpb.2015.12.008 26775139

[b8] Dilmac S. & Korurek M. ECG heart beat classification method based on modified ABC algorithm. Appl. Soft Comput. 36, 641–655 (2015).

[b9] Yu L. L., Tan B. X. & Meng T. X. The automatic classification of ECG based on BP neural network. Adv. Mat. Res. 121–122, 111–116 (2010).

[b10] Bhardwaj P., Choudhary R. R. & Dayama R. Analysis and Classification of Cardiac Arrhythmia Using ECG Signals. Int. J Comput. Appl. 38, 37–40 (2012).

[b11] Tantawi M. M., Revett K., Salem A. B. & Tolba M. F. A wavelet feature extraction method for electrocardiogram (ECG)-based biometric recognition. Signal Image Video. P. 9, 1271–1280 (2015).

[b12] Guler I. & Ubeyli E. D. ECG beat classifier designed by combined neural Networks. Pattern Recogn. 38, 199–208 (2005).

[b13] Jatmiko W., Nulad W. P., Elly M. I., Setiawan I. M. A. & Mursanto P. Heart Beat Classification Using Wavelet Feature Based on Neural Network. Wseas Trans. Syst. 10, 17–26 (2011).

[b14] Kumar R. G. & Kumaraswamy Y. S. Investigating Cardiac Arrhythmia in ECG using Random Forest Classification. Int. J Comput. Appl. 37, 31–34 (2012).

[b15] Ubeyli E. D. ECG beats classification using multiclass support vector machines with error correcting output codes. Digit. Signal Process. 17, 675–684 (2007).

[b16] Yu S. & Chou K. Integration of independent component analysis and neural networks for ECG beat classification. Expert. Syst. Appl. 34, 2841–2846 (2008).

[b17] Dutta S., Chatterjee A. & Munshi S. Correlation technique and least square support vector machine combine for frequency domain based ECG beat classification. Med. Eng. Phys. 32, 1161–1169 (2010). 20833096 10.1016/j.medengphy.2010.08.007

[b18] Javadi M., Ebrahimpour R., Sajedin A., Faridi S. & Zakernejad S. Improving ECG classification accuracy using an ensemble of neural network modules. Plos. One. 6, 1–13 (2011). 10.1371/journal.pone.0024386 PMC3202523 22046232

[b19] Ebahimzadeh A., Shkiba B. & Khazaee A. Detection of electrocardiogram signals using an efficient method. Appl. Soft. Comput. 22, 108–117 (2014).

[b20] Kutlu Y. & Kuntalp D. Feature extraction for ECG heartbeats using higher order statistics of WPD coefficients. Comput. Meth. Prog. Bio. 105, 257–267 (2012). 10.1016/j.cmpb.2011.10.002 22055998

[b21] Li H. Q. & Wang X. F. Detection of Electrocardiogram characteristic points using lifting wavelet transform and Hilbert transform. T. I. Meas. Control. 35, 574–582 (2013).

[b22] Li H. Q., Wang X. F., Chen L. & Li E. B. Denoising and R-Peak detection of electrocardiogram signal based on EMD and improved approximate envelope. Circ. Syst. Signal. Pr. 33, 1261–1276 (2014).

[b23] Moody G. B. & Mark R. G. The impact of the MIT-BIH arrhythmia database. IEEE Eng. Med. Biol. Mag. 20, 45–50 (2001). 11446209 10.1109/51.932724

[b24] Li H. Q. . A new ECG signal classification based on WPD and ApEn feature-extraction. Circ. Syst. Signal. Pr. 35, 339–352 (2016).

[b25] Holland J. H. Adaptation in Natural and Artificial Systems: an introductory analysis with applications to biology. Control & Artificial Intelligence. University of Michigan Press 6, 126–137 (1975).

[b26] Margarita R. G. A. & Christian G. Q. M. Using genetic algorithm feature selection in neural classification systems for image pattern recognition. Int. J. Mod. Phys. A. 33, 52–58 (2013).

[b27] Aslantas G., Gurgen F. & Salah A. A. GA-NN approach for ECG feature selection in rule based arrhythmia classification. Neural Netw. World. 24, 267–283 (2014).

[b28] Rai H. M., Trivedi A. & Shukla S. ECG signal processing for abnormalities detection using multi-resolution wavelet transform and artificial neural network classifier. J. Int. Meas. Confederation. 46, 2338–3246 (2013).

